# Vitamin E and selenium plasma concentrations in weanling pigs under field conditions in Norwegian pig herds

**DOI:** 10.1186/1751-0147-49-1

**Published:** 2007-01-03

**Authors:** Tore Sivertsen, Ellen Vie, Aksel Bernhoft, Børge Baustad

**Affiliations:** 1 Department of Production Animal Clinical Sciences, Norwegian School of Veterinary Science, P.O. Box 8146, 0033 Oslo, Norway; 2 National Veterinary Institute, P.O. Box 8156, 0033 Oslo, Norway; 3 Norwegian Pig Health Service, P.O. Box 8156, 0033 Oslo, Norway

## Abstract

**Background:**

The status of α-tocopherol (vit E) and selenium (Se) has been shown to influence disease resistance in pigs, and may be important for the health of weanling pigs.

**Methods:**

Plasma levels of both vit E and Se were followed in weanling pigs under field conditions in six Norwegian pig herds. Plasma vit E and Se were measured in 3 sows from each herd and 4 piglets in the litter of each sow at the day before weaning (day -1); and in the same piglets at days 4, 8 and 18 after weaning.

**Results:**

Mean plasma vit E was 4.0 μg/ml in the sows and 2.6 μg/ml in the piglets at day -1, fell to 1.6 μg/ml in the weanling pigs at day 4, and remained low. Mean plasma Se was 0.22 μg/g in the sows and 0.08 μg/g in the piglets at day -1, rose to 0.10 μg/g in the weanlings at day 4, and continued rising.

**Conclusion:**

The results suggest that vit E and Se supplementation to piglets and weanling pigs in Norway may still be suboptimal, but that levels of the two nutrients partially compensate for each other in the weaning period.

## Background

In feeding experiments with weanling pigs, a number of authors have observed a drop in vit E concentrations in the plasma of pigs in the first weeks after weaning [1-3]. Because vit E and Se status have been shown to influence immunological functions and disease resistance in pigs [4,5], these observations have attracted considerable attention in the pig industry. The fall in vit E has been related to low activity of carboxylester hydrolase enzymes in the gut of weanling pigs [6]. Because these enzymes cleave the dl-α-tocopheryl acetate form of the vitamin used in standard commercial feeds, the use of special supplements for weanling pigs containing non-esterified d-α-tocopherol has been advocated [7]. Other authors conclude that adequate plasma vit E levels in weanling pigs may be attained with sufficient dl-α-tocopheryl acetate in the feed [8].

In spite of the strong physiological interrelationship between vit E and Se, only some of the studies on post-weaning vit E levels have included Se measurements, and the Se status of the piglets has hardly been drawn into the discussion about additional vit E supplementation. Studies concentrating on Se only have found that transfer of Se from sows to piglets is limited [9], and that plasma Se concentrations in weanlings are highly dependant on Se levels in the post-weaning diet [10].

In Norway, as in most of Scandinavia, harvested grain is low both in Se and vit E [11]. However, most pigs are fed commercial diets, fortified with standardized amounts of vit E and Se. In the last decade, the use of specialized lactation and weaner diets has become widespread, and the amounts of vit E added to these feeds have gradually been increased [12]. Vit E supplements above 100 mg/kg are now common in feed for weanling pigs in Norway, in accordance with the recent recommendations of Moreira & Mahan [8] and others. On the other hand, the use of additional supplements with vitamins or minerals is uncommon [12].

In the present investigation we have examined the concentrations of both vit E and Se in the plasma of sows and piglets at weaning and in pigs in the first weeks after weaning, under field conditions in a number of representative Norwegian pig herds. The aim was to assess the adequacy of present feeding regimes in the Norwegian pig industry with respect to vit E and Se status for pigs in the weaning period.

## Materials and methods

### Herds and feeding

Six normal pig breeding herds were selected for the study. Only herds registered in In-Gris, the Norwegian litter recording system [13], were included. Batch organised farrowing was used in all the herds, all were fed standard commercial concentrates with no additional supplements, and all were located within 200 kilometres from Oslo. Within these criteria, the herds were selected mainly on the basis of practical considerations to make the collection of samples feasible. The pigs were Norwegian Landrace and hybrids with Large White, and the litters were of varying parity.

Information on feeding and management in the selected herds was recorded, but no changes were done to the choice of feed or the normal feeding and management routines in the herds in relation to the study. Commercial diets for breeding sows and/or weanling pigs were used in all the herds. According to the feed labels, the feeds used both for the sows and the weanling pigs were supplemented with 0.30–0.40 mg Se per kg, in the form of sodium selenite; and 110–161 mg vitamin E per kg, as α-tocopheryl acetate.

### Design and sampling

In each herd, one farrowing group of sows with litters scheduled for weaning in January or February 2003 was chosen. Within each of these groups, three sows with litters were selected at random, and the farrowing and weaning days for each litter recorded. For the selected litters, the average age at weaning was 37.2 days, with standard deviation 5.6 days and range 32–49 days. At the day before weaning (day -1) blood samples were drawn from the sows and five piglets in each litter. In selecting the piglets for sampling, an even sex distribution within each litter group was sought. The sampled piglets were marked individually, and their body weights recorded. At days 4, 8 and 18 after weaning, blood samples were again drawn from the marked pigs, and new body weights recorded. The original aim was to secure a full sample set from 3 pigs in each litter, taking into account that some piglets would die in the period. However, the loss of weanling pigs in the selected litters was small, so none of the litters ended up with less than 4 full sets. In those litters having 5 full sets, one sample set from the most numerous sex was chosen at random and discarded. All samples were drawn in 10 ml heparinized Vacutainer^® ^tubes.

### Chemical analysis

In the laboratory, a few ml of blood was taken out from each sample before centrifugation, and stored separately. All blood and plasma samples were stored at -20°, and analysed within 3 months. Vit E (α-tocopherol) and Se concentrations were determined in all 306 plasma samples. In addition, Se concentrations in whole blood were determined in samples from one sow from each farm, and in all samples from two of her piglets; 54 samples altogether.

Vitamin E concentrations were determined with HPLC. After precipitation of proteins with ethanol (+1% ascorbic acid), tocopherols in the plasma samples were extracted with hexane. The hexane was evaporated and the residue redissolved in ethanol. Vit E concentrations were measured with a Perkin-Elmer HPLC unit, using a Supelguard C18 aptron precolumn, a reversed-phase C18 separation column with a methanol-water (95/5) mobile phase, and a Shimadzu fluorescence detector. Calculations were based on the use of tocol as an internal standard in all samples. The recovery of tocol was within 90–110%. Detection limit for α-tocopherol was 0.1 μg/ml.

Selenium concentrations were determined by atomic absorption spectrometry with a hydride generator system [14], using a Varian SpectrAA-30 with a VGA-76 vapour generation accessory. Before analysis, each sample was prepared by oxidative digestion in a mixed solution with concentrated nitric and perchloric acids, using an automated system with a Tecator 1012 Controller and 1016 Digester heating unit. The method is accredited (NS-EN ISO/IEC 17025). A quality control system using regular analyses of a blood standard (proficiency tested pig blood 1992) with value 0.20 ± 0.02 μg/g was adopted as reference material. During the project period, the measured concentration of the reference material was 0.19 ± 0.01 μg/g (N = 17). For practical reasons, the National Veterinary Institute calculates plasma and blood Se concentrations in μg/g. 1 μg/g blood corresponds to 1.052 μg/ml, and 1 μg/g plasma to 1.026 μg/ml [15]. The detection limit was 0.01 μg/g.

### Statistical calculations

All correlations were evaluated with Pearson correlation analysis. To evaluate the temporal changes in plasma vit E and Se concentrations in the weanling pigs statistically, a regression model analysis was used, with pig number as a cluster variable to adjust for repeated measurements (Stata for Windows, SE 8.2, Stata Corp. College Station, TX; regress procedure). The correlation structure between measurements and the degree of variance at individual and litter level was tested using the xtgee procedure in Stata, which allows for incorporating the correlation structure in a repeated measurement model. Model assessment was done by comparing estimates from the regression model and the xtgee procedure, and by examining predictions and residuals by x-y plots and normal quantile plots. In addition, the statistical differences between the average Vit E and Se concentrations at day -1 and day 4 were analysed for each litter, with student's t-test (paired analysis).

The relative contributions of farm and litter to the total variance of plasma vit E, plasma Se concentration and body weight at day -1 were evaluated with variance component analysis, using SAS statistical software (ver. 8, SAS institute inc.). The same method was used to evaluate the relative contributions of farm and litter to the variance in the size of plasma vit E and Se changes from day -1 to day 4.

Because herd 1 differed significantly from all the other herds in age and bodyweight at weaning, both the analysis of correlations with age at weaning and the variance component analysis were done twice; with and without herd 1 included.

## Results

Mean values for plasma vit E, plasma Se and body weight in all sows and weanling pigs for each sampling day are shown in Table 1, with standard deviations and ranges.

**Table 1:** Plasma vit E and Se in sows and weanling pigs – summary table

		Sows (n = 18)	Weanlings (n = 72)			
Sampling day:		Day -1	Day -1	Day 4	Day 8	Day 18
Vit E (μg/ml)	Mean; std dev	4.0 (1.0)	2.6 (0.8)	1.6 (0.6)	1.5 (0.5)	1.7 (0.7)
	Range	1.8–5.6	0.7–4.7	0.4–4.1	0.4–2.6	0.7–3.6
Se (μg/g)	Mean; std dev	0.22 (0.02)	0.08 (0.01)	0.10 (0.01)	0.11 (0.01)	0.12 (0.01)
	Range	0.18–0.26	0.05–0.11	0.08–0.13	0.07–0.13	0.09–0.15
Body weight (kg)	Mean; std dev		11.9 (4.0)	12.8 (4.3)	14.2 (4.7)	19.2 (6.1)
	Range		4.9–22.3	3.7–24.0	5.5–27.1	8.6–35.9

Plasma vit E (α-tocopherol) and plasma Se in sows and weanling pigs, and bodyweight in weanling pigs, in six Norwegian herds in relation to weaning. Means, standard deviations (in brackets), and ranges. Day 0 is the day of weaning.

**Figure 1:**
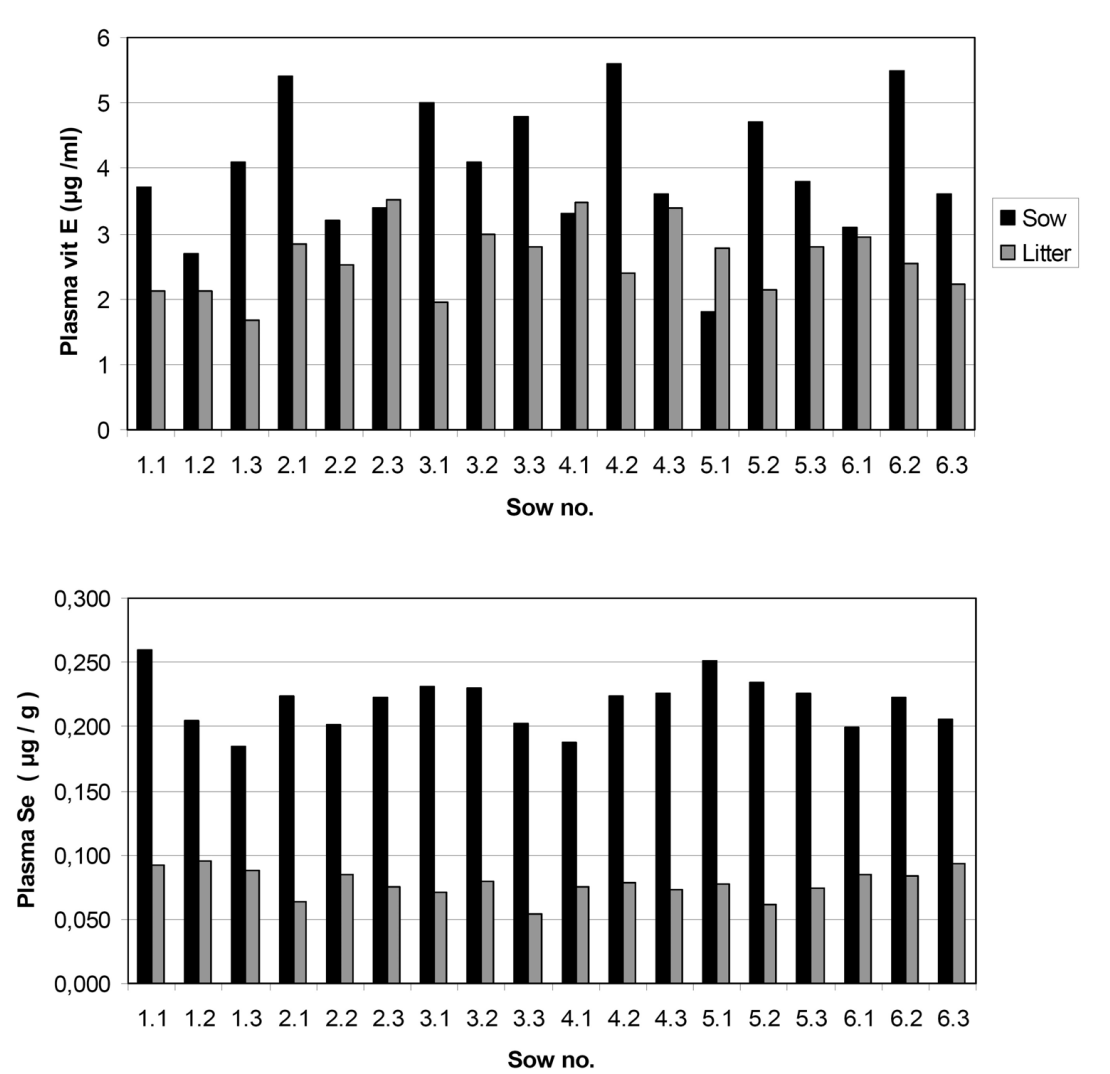
**Plasma vit E and Se in sows and their litters at weaning**. Plasma vit E and Se concentrations in sows and average concentrations in their litters, at the day before weaning (day -1), in six Norwegian pig herds.

In Fig. 1, the relation between plasma vit E and Se concentrations in each sow and the average level in her litter at day -1 is illustrated. No significant correlation was found between plasma concentrations in the sows and the average plasma concentrations in their litters at day -1, neither for vit E nor for Se. In the entire data set, plasma vit E in the piglets at day -1 was negatively correlated to age at weaning (r = -0.40), while both plasma Se (r = 0.38) and bodyweight (r = 0.79) were positively correlated to weaning age. When herd 1 was excluded from the analysis, only the correlation between bodyweight at day -1 and weaning age remained statistically significant (r = 0.35, p < 0.01).

Mean plasma vit E concentrations in the weanling pigs fell substantially from day -1 to day 4, and stayed low for the rest of the period. Plasma Se concentrations increased from day -1 to day 4, and continued rising slightly till the end of the study (Table 1). In the regression model analysis for the temporal changes in plasma vit E and Se, no specific correlation pattern between repeated measurements was found, and thus a simple cluster adjustment for the repeated measurement was used, by implementing the robust estimator in the regression model procedure. The final model for both vit E and Se included day post weaning and herd as explanatory variables, while litter, individual age and weight were dropped. Regression diagnostics did not reveal any serious deviations, model fit was acceptable, and estimates from the regression model and the xtgee analysis showed only marginal differences. Clear and significant differences between day -1 and each of the days after weaning were shown for both plasma Se and plasma vit E. However, the 95% confidence intervals indicated that the model predictions for plasma vit E at day 4, day 8 and day 18 were not significantly different, while the differences between these three days for plasma Se were clearly significant.

**Figure 2:**
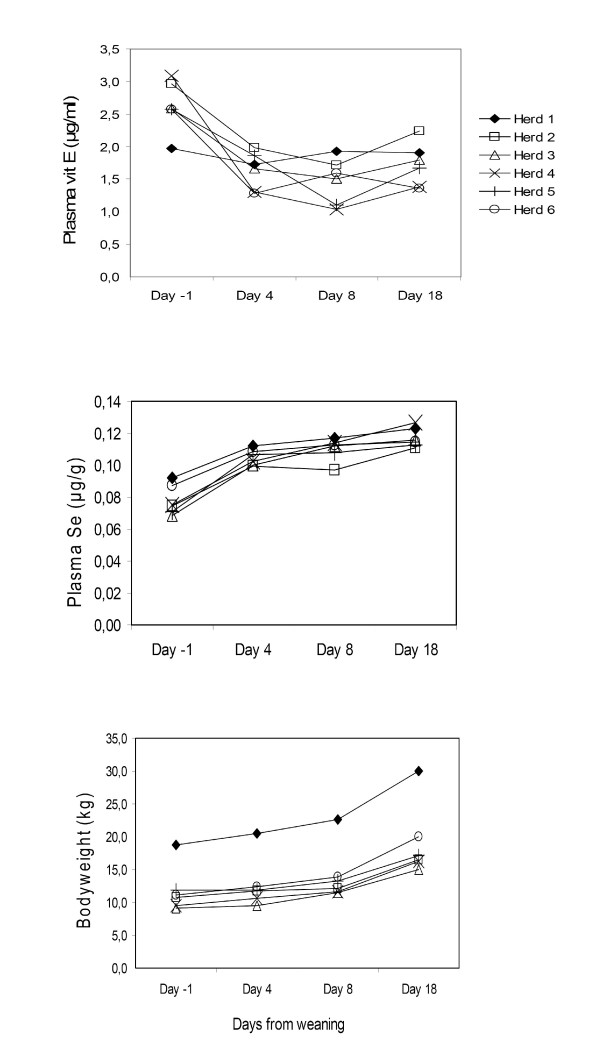
**Changes in plasma vit E, plasma Se and bodyweight in each herd**. Changes in average plasma vit E, average plasma Se and average bodyweight in weanling pigs from the day before weaning to day 18 post weaning, in each of the herds studied.

In Fig. 2, the development in mean plasma vit E, mean plasma Se and mean body weight is shown for each herd. Analysed litter by litter, the fall in plasma vit E from day -1 to day 4 was statistically significant by student's t-test (paired analysis) in 10 of the 15 litters in herds 2 to 6, but not in any of the litters in herd 1. The rise in plasma Se from day -1 to day 4 was statistically significant in 16 of the 18 litters studied. The average daily weight gain in all pigs from day -1 to day 18 was 384 g/day, with standard deviation 147 g/day and range 147 to 753 g/day. Throughout the study, mean body weight in herd 1 was significantly higher than in all the other herds.

Results of the variance component analysis are shown in Table 2, with and without herd 1 included.

**Table 2:** Relative contribution of farm and litter to variation in plasma vit E and Se

All herds:						
Variable:	Vit E		Se		Bodyweight	
	At D -1	D -1 to D 4	At D -1	D -1 to D 4	At D -1	Daily gain

Source of variation:						
Farm	12.5%	25.7%	32.0%	45.6%	66.7%	58.2%
Litter	13.7%	0.4%	18.0%	33.4%	9.4%	2.3%
Remnant variance	73.7%	73.8%	50.0%	21.0%	23.9%	39.4%

Herds 2 to 6:						

Variable:	Vit E		Se		Bodyweight	
	At D -1	D -1 to D 4	At D -1	D -1 to D 4	At D -1	Daily gain

Source of variation:						
Farm	2.8%	15.9%	16.6%	11.4%	8.3%	31.9%
Litter	20.2%	3.4%	32.6%	22.7%	26.1%	7.1%
Remnant variance	77.0%	80.6%	50.7%	65.8%	65.7%	61.0%

Variance component analysis of variation in the following variables of plasma vit E, plasma Se and bodyweight in weanling pigs: Start values at day -1 (At D -1), changes from day -1 to day 4 (D -1 to D 4), and daily bodyweight gain from day -1 to day 18 (Daily gain). Relative contribution from different sources of variation to the total variance of each variable, with and without herd 1 included.

In the 6 sows analyzed, the mean whole blood Se concentration at day -1 was 0.20 μg/g, and the average ratio plasma Se/blood Se was 1.07. In the 12 weanling pigs, the average ratio plasma Se/blood Se was calculated for each sampling day, and varied between 0.83 and 0.91. There was a strong correlation between plasma Se and blood Se in the weanling pigs (r = 0.90, p < 0.0001), but much weaker correlation in the sows (r = 0.49, n.s.).

## Discussion

The mean plasma concentration of vit E in piglets at day -1 in this study was 2.6 μg/ml, or about 65% of the mean plasma level in their sows. This relative ratio between piglet and sow plasma levels at weaning is in line with findings in the feeding study of Håkansson et al. [2]. The plasma vit E level at day -1 was highly variable, both in sows and in their piglets (Fig. 1), but there was not statistically significant correlation between plasma vit E in the sows and in their piglets. This seems surprising, but is compatible with the finding of Håkansson et al. [2] that plasma vit E levels in piglets, both at 3 days and 5 weeks of age, were primarily correlated to the vit E levels in colostrum.

The observed fall in plasma vit E after weaning is in line with the results reported by several authors [2,3,6,16]. The regression model analysis did not show any significant differences in plasma vit E between the three sampling days after weaning. The apparent turn in the mean plasma vit E level around day 8 (Table 1) may therefore be fortuitous, or it may reflect a real trend that could not be confirmed statistically due to the limited size of the study. Håkansson et al. [2] found a similar reversion, with a rise in plasma vit E levels from one week to four weeks after weaning. The fall in plasma vit E from day -1 to day 4 was confirmed in 5 of the 6 herds in this study. In herd 1, however, the mean vit E level changed very little after weaning (Fig. 2). In this herd, the piglets were uncommonly old (48 days) and well developed at weaning, as reflected in their bodyweights. Possibly, these pigs had already started eating some of the sow's feed, and may have been "half-weaned" already at day -1. However, the possibility that the difference may also reflect age-related physiological changes in vit E absorption cannot be excluded. In herds 2–6 the average weaning age was 34.9 days. The average weaning age in the entire In-gris litter recording system in the same year was 35.0 days [13].

When herd 1 was excluded, differences between litters contributed more to the total variation of plasma vit E at weaning in the variance component analysis than the herd differences did (Table 2). This indicates a certain effect of the sows on piglet plasma vit E levels, in spite of the lack of correlation between litter and sow plasma levels at weaning. In the variation of the change in plasma vit E from day -1 to day 4, however, the litter effect was smaller than the effect of herds, even with herd 1 excluded. This may imply that feeding and management factors in the herds influence the size of the fall in plasma vit E after weaning more strongly than they influence the plasma level at weaning.

The mean plasma Se concentration of the piglets at day -1 was 0.08 μg/g, or 36% of the mean plasma Se in the sows. This result is quite similar to that observed by Mahan & Moxon [10] with sows fed a diet supplemented with 0.1 mg Se/kg. The individual variation in plasma Se concentrations at weaning was much smaller than for vit E (Fig. 1). This may partially explain the lack of significant correlation between plasma Se in the sows and their litters. The significant rise in plasma Se from day -1 to day 4, and the further, more limited rise throughout the observation period, are in line with the findings of Mahan & Moxon [10] in weaned pigs fed a diet supplemented with 0.3 mg Se/kg. As for vit E, the individual variation between the pigs dominated over litter or herd effects both in the regression model and in the variance component analysis. For the Se variables, however, the contribution to the total variation was more evenly distributed between herd and litter effects, though litter effects were somewhat more important when herd 1 was excluded (Table 2).

The analysis of whole blood Se in a number of samples was done to control for possible variation between plasma and whole blood Se concentration. Because of the high amount of glutathione peroxidase in erythrocytes, analysis of Se in whole blood is relevant to Se status [17], and may differ from the plasma values. In this study, the correlation between whole blood and plasma Se was very high in the piglets, but weaker in the sows.

There is no general agreement in the literature on limits between adequate, marginal and deficient plasma levels in pigs, neither for vit E nor for Se. In Norway, the National Veterinary Institute has considered plasma levels of vit E below 1.0 μg/ml as distinctly deficient, while the Danish Institute of Agricultural Sciences usually considers plasma vit E concentrations below 1.5 μg vit E/ml to indicate a state of deficiency [18]. At day -1, only 7% of the piglets had plasma concentrations of vit E below 1.5 μg/ml. In the period from day 4 to day 18, however, the proportion of pigs with plasma vit E below 1.5 μg vit E/ml varied between 39 and 56%. At day 8, 17% of the pigs had plasma vit E concentrations that were even below 1.0 μg vit E/ml.

For selenium, Ullrey [19] and Mahan [20] consider serum concentrations from 0.08 to 0.15 ppm Se as normal in pigs, while Blood & Radostits [21] declare above 0.120 μg/ml serum as normal levels, and 0.005 to 0.060 μg/ml as deficient. In the present study, 54% of the piglets had plasma Se concentrations below 0.08 μg/g at day -1, and 19% had concentrations at 0.06 μg/g or less. From day 4 to day 8, however, only 1–3% of the pigs had plasma Se concentrations below 0.08 μg/g, and at day 18 no pigs were found to have Se concentrations below this limit.

The present study includes a limited number of pig herds, but with the exception of herd 1 with its unusually high weaning age, we consider the herds included to be fairly representative for pig herds registered in the Norwegian In-Gris system. The In-Gris litter recording system includes the majority of pig breeding units in Norway, with 70.1% of all breeding sows in 2002 [13]. For herds 2 to 6, the results of this study were quite consistent, with limited variation both between and within herds. In our opinion, our results do therefore suggest that a large proportion of Norwegian piglets under present feeding conditions has acceptable vit E levels, but marginal Se levels in plasma at weaning. They also suggest that plasma vit E levels drop to marginal or deficient levels in a large part of Norwegian weanling pigs in the first weeks post-weaning, while plasma Se in the same period seems to rise to acceptable levels. It must be emphasised that our results are only representative for healthy weanling pigs. In pigs that develop diarrhea around weaning vitE absorption may be significantly reduced [22].

The clinical significance of these findings is more difficult to assess. In feeding trials with different vit E and Se levels, effects on daily weight gain and other performance parameters are generally not observed [8]. No special disease problems were seen or reported by the participating farmers in the present study. Cardiac and skeletal muscle degeneration and liver necrosis are the most typical and dramatic disease conditions seen in vit E and Se deficient pigs [23]. In Norway these conditions usually affect older pigs, and they have become less frequent after Se supplementation was introduced in 1980 [24]. The most important consideration may be whether marginal vit E and Se levels around weaning affect the disease resistance in the pigs. Vit E and Se deficient pigs may have impaired immune responses [5,25], and have been shown to be more susceptible to swine dysentery [4]. Weaning is a critical time for the young pigs, and post-weaning diarrhoea and other weaning-related diseases are important health problems.

The fall in plasma vit E after weaning observed in feeding trials has led to considerable interest for the forms of vit E used in feed supplements. Some of the fall may be related to a lower intestinal carboxyl ester hydrolase activity in pigs in the post-weaning period [26]. When given in equal amounts, the natural d-α-tocopherol leads to higher plasma vit E levels than the more stable dl-α-tocopheryl acetate normally used in pig feeds [1]. However, most of this difference may be explained by the difference in biological activity between d-α-tocopherol and the racemic dl-α-tocopherol [27]. While some authors strongly advocate the use of additional supplements with d-α-tocopherol in its alcohol form [7], others have found that the post-weaning fall in plasma vit E may be counteracted by increasing the levels of dl-α-tocopheryl acetate in the feed ration [8,16]. Because of the functional interrelationship between vit E and Se, another possible approach to protect the health of weanling pigs is to try to increase the Se levels in piglets before weaning. A further increase in the Se level fed to the sows is probably neither efficient [9] nor practicable, as feed regulations in the EU and the EEC set an upper total limit in the feed at 0.5 mg Se/kg [28]. Thus, other ways of supplementing piglets with Se in the nursing period may be worth consideration.

## Conclusion

In conclusion, our results suggest that the vit E and Se status in the weaning period may still be suboptimal for a large proportion of pigs with present feeding regimes in Norway, but that the plasma levels of the two nutrients may partially compensate for each other, as they tend to change in opposite directions in the first weeks post-weaning. In our opinion, the results warrant a renewed evaluation of the amounts and methods used for supply of both vit E and Se to piglets and weanling pigs in Norway.

## Competing interests

The two companies that have supported this study economically are both involved in the marketing of vitamins and feed additives to pigs in Norway. Neither of them has been involved in the planning or performance of the study or in the interpretation of the results. None of the authors are affiliated to these companies in any other ways.

## Authors' contributions

TS and BB conceived of the study, and TS is the main author of the paper. EV did the main part of the practical work; both with blood sampling, chemical analysis, primary analysis of the results and first preparation of tables and figures. AB was responsible for the chemical work and was the main tutor for EV, especially in the chemical and analytical part. BB selected the pig herds and did most of the arrangements with the farmers. All authors have to some extent participated both in the planning, the practical work and the interpretation of the results, and all authors have read and approved the final manuscript.
